# The use of genotoxicity biomarkers in molecular epidemiology: applications in environmental, occupational and dietary studies

**DOI:** 10.3934/genet.2017.3.166

**Published:** 2017-08-11

**Authors:** Carina Ladeira, Lenka Smajdova

**Affiliations:** 1 Environment and Health Research Group, Escola Superior de Tecnologia da Saúde de Lisboa-Instituto Politécnico de Lisboa (ESTeSL–IPL), Av. D. João II, Lote 4.69.01, 1990-096 Lisboa, Portugal; 2 Grupo de Investigação em Genética e Metabolismo, Escola Superior de Tecnologia da Saúde de Lisboa-Instituto Politécnico de Lisboa (ESTeSL–IPL), Av. D. João II, Lote 4.69.01, 1990-096 Lisboa, Portugal; 3 Centro de Investigação em Saúde Pública-Escola Nacional de Saúde Pública, (CISP-ENSP), Universidade Nova de Lisboa, Portugal; 4 Faculty of Social Sciences, London Metropolitan University, London, United Kingdom

**Keywords:** molecular epidemiology, biomarkers, genotoxicity, micronuclei, comet assay, environment, occupation, diet

## Abstract

Molecular epidemiology is an approach increasingly used in the establishment of associations between exposure to hazardous substances and development of disease, including the possible modulation by genetic susceptibility factors. Environmental chemicals and contaminants from anthropogenic pollution of air, water and soil, but also originating specifically in occupational contexts, are potential sources of risk of development of disease. Also, diet presents an important role in this process, with some well characterized associations existing between nutrition and some types of cancer. Genotoxicity biomarkers allow the detection of early effects that result from the interaction between the individual and the environment; they are therefore important tools in cancer epidemiology and are extensively used in human biomonitoring studies. This work intends to give an overview of the potential for genotoxic effects assessment, specifically with the cytokinesis blocked micronucleus assay and comet assay in environmental and occupational scenarios, including diet. The plasticity of these techniques allows their inclusion in human biomonitoring studies, adding important information with the ultimate aim of disease prevention, in particular cancer, and so it is important that they be included as genotoxicity assays in molecular epidemiology.

## 1. Introduction

Genetic factors are clearly important in terms of influencing individual susceptibility to carcinogens; however, external factors represent the greatest opportunity for primary prevention. By ‘external factors’ we mean those related with environment—a broad scope, including all non-genetic factors such as diet, lifestyle and infectious agents. In a more specific approach, environmental factors include natural or man-made agents encountered by humans in their daily life, upon which they have no or limited personal control. The most important ‘environmental’ exposures, defined in this strict sense, include outdoor and indoor air pollution and soil and drinking water contamination [1]. In a more specific environmental niche are the occupational settings. People who work in certain jobs may have a higher risk of cancer due to exposure to some chemicals, radiation, or other aspects of their work (ergonomics, complex networks of safety risks, and many and varied psychosocial factors). Activities such as agriculture, painting, and industry are examples where workers can handle certain chemicals or be exposed to hazardous agents that can increase the risk of developing cancer [2]. Diet is also included in environment, particularly in lifestyle, and recognition of its importance has increased in recent decades, since it is a factor linked to some types of cancer [3],[4]. The molecular epidemiology approach, measuring molecular or cellular biomarkers as indicators of disease risk or exposure to causative or preventive factors, has applications in studies of environmental and occupational exposure, disease etiology, nutrition, lifestyle and others [5], particularly in biomonitoring of populations.

This review aims to demonstrate the importance of genotoxicity biomarkers, such as those provided by cytokinesis blocked micronucleus assay and comet assay, as molecular epidemiology tools in human biomonitoring studies. With this approach, it is possible to detect, and therefore, prevent disease, specifically cancer in a wide variety of exposures—environmental, occupational and from diet.

## 2. Molecular Epidemiology

Classical epidemiology has historically been the hallmark approach to demonstrate associations between exposure to hazardous substances and development of disease; however, inter-individual variation, i.e., genetic/individual susceptibility, did not have a place in this equation. The development of molecular biology and its use as a potential tool in epidemiological studies strengthened the identification of diseases associated with environmental exposures related to lifestyle, occupation, or ambient pollution. In molecular epidemiology, laboratory methods are employed to document the molecular basis and preclinical effects of environmental carcinogenesis [6]–[9].

Molecular epidemiology has the advantage of being directly relevant to human risk, unlike animal or other experimental models that require extrapolation to humans. Moreover, biomarker data on the distribution of procarcinogenic changes and susceptibility factors in the population can improve the estimation of cancer risk from a given exposure [10]. Increasingly, molecular epidemiology studies are incorporating panels of biomarkers relevant to exposure, preclinical effects and susceptibility, using blood and exfoliated cells, tissues and body fluids. These biomarkers are now being widely used in cross-sectional, retrospective, prospective and nested case-control epidemiologic studies, with the aim of improving our understanding of the causes of specific human cancers [5],[11].

It is well established that maintaining the integrity of the genome is essential for normal cell function and any disruption in the process can lead to either cell death or cancer development [12], and so the majority of the available biomarkers used in molecular epidemiology studies are related to agents that cause DNA damage and are mutagenic [5],[13]. Major gains in cancer prevention should stem from theoretically important strategies, namely regulations, public education programs, health surveillance, behavior modification, and chemoprevention programs and other interventions that adequately protect these groups from environmental carcinogens [10],[14].

## 3. Biomarkers of Genotoxicity

Traditionally, biomarkers are defined as biomarkers of exposure, effect and individual susceptibility. For the purpose of this review, we will focus on biomarkers of effect. A biomarker can be any substance, structure or process that can be monitored in tissues or fluids and that predicts or influences health; or that assesses the incidence or biological behavior of a disease, but is not a measure of disease, disorder or health condition itself [15],[16]. Ideally, biomarkers should be accessible (non-invasive), non-destructive, easy and cheap to measure [17],[18].

One of the criteria for establishing associations between an exposure and disease is biological plausibility. In this context, biomarkers may contribute by illuminating some of the carcinogenic steps linked to a particular risk factor. This is possibly an undervalued area where biomarkers can make significant contributions to cancer epidemiology. If a particular chemical exposure from ambient air is associated with increased risk, the additional information that exposed individuals have higher levels of DNA damage would add support to the exposure-disease association [19].

Biomarkers of effect offer the opportunity to provide scientific confirmation of proposed exposure-disease pathways in human populations, since they can be elicited as a result of interaction of the biological system with the environment [20],[21]. The increasing demand for information about health risks derived from exposure to complex mixtures calls for the identification of biomarkers to evaluate genotoxic effects associated with occupational and environmental exposure to chemicals, and other potential sources of damage. An important group of effect biomarkers are genotoxicity biomarkers, which have been developed in vitro (cells and cell lines), in vivo (animals) and ex vivo (cells from humans). Cytogenetic biomarkers are the most frequently used endpoints in human biomonitoring studies, and are extensively used to assess the impact of environmental, occupational and other factors in genetic (in)stability [20]–[22]. Among the wide range of cytogenetic biomarkers, micronuclei in lymphocytes provide a promising approach to assess health risks [23].

The most used biological matrices for studying genotoxic effects in human biomonitoring are blood lymphocytes and exfoliated cells, both being easy to sample. Lymphocytes circulate throughout the body, have a reasonably long life span, and can therefore be damaged in any specific target tissue by a toxic substance [24]. Exfoliated buccal cells have been effective in showing the genotoxic effects of lifestyle factors such as tobacco smoking, alcohol, medical treatments, such as radiotherapy as well occupational and environmental exposure, namely exposure to potentially mutagenic and/or carcinogenic chemicals, and in studies of chemoprevention of cancer (antioxidants) and evaluation of malignant transformation of preneoplastic lesions associated with oral squamous cell carcinoma [25]–[33].

### 3.1. Cytokinesis Blocked Micronucleus (CBMN) Assay

Living organisms may be exposed to mutagenic substances that cause cellular damage, which may be induced by chemical, physical or biological agents that affect DNA, chromosome replication and gene transcription, causing abnormalities that may lead to cancer and cell death [34].

The cytokinesis-blocked micronucleus (CBMN) assay is a comprehensive system for measuring DNA damage, cytostasis and cytotoxicity-DNA damage events scored specifically in once-divided binucleated cells. It is a method for assessing DNA damage caused by xenobiotics, allowing detection of effects caused by clastogenic agents (that provoke chromosome breakage) and aneugenic agents (abnormal chromosome segregation associated with loss) [34]–[38]. Other endpoints that can be measured are nucleoplasmic bridges (NPB), a biomarker of DNA misrepair and/or telomere end-fusions, and nuclear buds (NBUD), a biomarker of elimination of amplified DNA and/or DNA repair complexes [29],[39].

The CBMN assay is regularly used as an in vitro test in genotoxicity testing (OECD 487) and it is the preferred method in human biomonitoring studies to detect cytogenetic effects after exposure to genotoxic agents. It is regarded as an indicator of mutagen sensitivity, a biological dosimeter of ionizing radiation exposure, a measure of DNA-repair capacity and genomic stability, and a predictor of cancer susceptibility/risk [40],[41]. In summary, it is defined as a robust assay for genetic damage with applications in ecotoxicology, nutrition, radiation sensitivity testing both for cancer risk assessment and optimization of radiotherapy; as well as these applications in biomonitoring of human populations, it is important for testing new pharmaceuticals and other chemicals. There are expectations regarding the future development of an automated system that can reliably score the various endpoints which are possible with the CBMN assay [29].

### 3.2. Comet Assay

The comet assay (otherwise called single-cell gel electrophoresis—SCGE) is a simple, sensitive method for detecting DNA-strand breaks. DNA strand breaks can originate from the direct modification of DNA by chemical agents or their metabolites; from the processes of DNA excision repair, replication, and recombination; or from the process of apoptosis. Direct breakage of the DNA strands occurs when reactive oxidative species (ROS) interact with DNA. Alkali-labile sites generated by loss of bases in the DNA, are converted to strand breaks by alkaline treatment (pH above 13.1) and so are also detected with the comet assay [42].

This assay was adapted to measure oxidized purines and oxidized pyrimidines by incubation of the nucleoids (the DNA structures remaining after lysis of agarose-embedded cells) with bacterial DNA repair enzymes [43] including formamidopyrimidine DNA glycosylase (Fpg), which recognizes the oxidized purine 8-oxoguanine, one of the most studied molecules regarding oxidative damage [34],[43].

Comet assay has become one of the standard methods for assessing DNA damage, with a wide range of applications, namely in genotoxicity testing, human biomonitoring and molecular epidemiology, ecogenotoxicology (monitoring environmental pollution by studying sentinel organisms), research on oxidative damage as a factor in disease, monitoring oxidative stress in animals or human subjects resulting from exercise, or diet, or exposure to environmental agents as well as fundamental research in DNA damage and repair [9],[44]–[46].

The congruence of results between the comet assay and other endpoints such as micronuclei or sister chromatid exchanges (SCE), has been one of the principal reasons to increase the use of the comet assay as a biomarker for hazard assessment, particularly in monitoring the effects of occupational hazards [47]–[52].

## 4. Human Genome-Environment Interaction—Biomonitoring as a Tool

The relative contribution of genetics versus the environment to human illness has been debated for decades, as the so-called gene-environment interaction. The importance of environmental exposures has been supported by geographic differences in incidence of disease, by variation in incidence trends over time, and by studies of disease patterns in immigrant populations [53].

Understanding risks to human health in the light of human genome-environment interactions is one of the most compelling challenges in environmental public health. With the sequencing of the human genome, renewed interest in understanding the role of the environment as a cause of human disease has emerged. Genes are expressed in response to the environment [54] and there are two kinds of susceptibility genes: those that predispose to disease without exposure to environmental factors and those that increase risk only by interaction with environmental agents [53]. Information about environmental risk factors should point to genes that might modify the risk, and identification of susceptibility genes should help identify previously unrecognized environmental risk factors [53].

Human biomonitoring has tremendous utility providing an efficient and cost-effective means of measuring human exposure to hazardous substances establishing evidence that both exposure and uptake have been taking place [55],[56]. This approach considers all routes of uptake and all sources which are relevant, making it an ideal instrument for risk assessment and risk management. It can identify new chemical exposures, trends and changes in exposure, establish distribution of exposure among the general population, identify vulnerable groups and populations with higher exposures, and identify environmental risks at specific contaminated sites with relatively low expenditure [56]. More attention should be given to monitoring populations which are known to be exposed to hazardous environmental contaminants and to providing reliable health risk evaluation, since that information is useful for supporting regulations on protection of the environment [57].

There are well-established national human biomonitoring survey programs worldwide, where a target population has been identified, questionnaires have been developed and sample collections have taken place. In Europe there are the German Environmental Survey (GerES, Germany), the Flemish Environment and Health Study (FLEHS, Belgium), the French National Survey on Nutrition and Health (ENNS, France), BIOAMBIENT.ES (Spain), Program for Biomonitoring the Italian Population Exposure (PROBE, Italy), Human Biomonitoring Project (CZ-HBM, Czech Republic). In America there are the Canada Health Measures Survey (CHMS) and the United States of America the National Health and Nutrition Examination Survey (NHANES), and in Asia, the Korea National Survey for Environmental Pollutans in the Human Body (KorSEP).

## 5. Environmental Exposure

Nowadays people have to suffer the mutagenic and carcinogenic effects of many genotoxic agents in daily life and working environments due to changing lifestyles and innovations, for instance, chemical substances such as drugs, food additives, pesticides, and nanomaterials [58].

Anthropogenic pollution has become inherent to the modern environment. The global and rapid increase in technogenic stress in the biosphere raises the question about possible consequences for biota, including man, acknowledging that all forms of life are inter-connected and that human health is strongly linked to the ecosystem's health [59]. Environmental chemicals and contaminants are ubiquitous, occurring in water, air, food and soil. While some chemicals are short-lived in the environment and may elicit no harmful effects in humans, other chemicals bioaccumulate or persist for a long time in the environment or the human body due to frequent exposure, potentially leading to adverse health effects [60].

A more integrated approach is needed to deal with the fact that adverse biological effects induced by exposure to complex pollutant mixtures are not easily interpreted from a set of chemical analyses. The toxic effect of different interacting pollutants can be either additive, synergistic or antagonistic [61]. Molecular epidemiology studies on populations environmentally or occupationally exposed to high levels of complex mixtures of urban air pollutants have revealed genotoxic effects in terms of increased incidence of DNA damage [5],[62]. Atmospheric pollutants, such as carbon monoxide, ozone, nitrogen oxides, sulfur dioxide, polycyclic aromatic hydrocarbons, and particulate matter are examples of chemical agents that may lead to DNA damage [34] and pose a serious threat to the health and the well-being of humans. According to their physicochemical properties, for instance, polycyclic aromatic hydrocarbons (PAHs) are released into the environment from both natural and anthropogenic sources, and are highly mobile in the environment, allowing them to distribute across air, soil, and water, becoming effectively ubiquitous [63],[64]. It is also of great importance to assess the risk of future health effects from accidental or occupational radiation exposure to humans in order to be able to take appropriate measures to protect exposed individuals [65]. Multidisciplinary approaches combining chemical, ecotoxicological and ecological data have been undertaken to develop effective methods for assessing the quality of the environment, identifying the extent of genetic changes that occur when organisms are exposed to chronic, low-level, anthropogenic pollutants in selected species, such as protozoa, dicotyledonous plants [61], *Scots pine* [59], invertebrate and vertebrate native marine species [66], and others.

It is important to note that the genotoxicity biomarkers are applied in ecotoxicological studies; moreover, the application of early warning (sublethal) biomarkers in water-river quality monitoring programs is highly recommended since some of the pollutants are also relevant from a human health perspective—causing endocrine disruption, immune responses, or genotoxicity [61]. However this paper will cover just the effects in humans and human cells. Table 1 summarizes some studies regarding to environmental exposure, namely air pollutants [67]–[69], heavy metals [70],[71], herbicides [72], mobile radiation [73], pesticides [74],[75], pollution mixture [76], PAHs [77],[78], and pyrethroids [79].

**Table 1.** Studies of human populations related environmental exposures.

Risk factor/exposure	Studied population/number of samples/sample	Genotoxicity biomarkers	Results				Refs.
Air pollutants (CO, NO_2_, SO_2_, benzene, O_3_, PM10 and PM2.5)	Children (Northen Italy)/N = 181/exfoliated buccal cells	MN assay	MN mean ± SD: 0.29 ± 0.13. MN mean frequency of 0.29%: 2–3-fold higher than that considered as a “reference” value for children of this age.				[67]
Air pollutants: domestic heating (SO_2_ and PM); traffic (NO_x_ VOCs)	Children (suburban, urban-traffic sites in Turkey)/N = 1.841 summer; N = 1.497 winter/buccal epithelial cells	MN assay		MN (‰) (mean ± SD)	BEC with MN (‰) (mean ± SD)		[68]
			Summer period	2.73 ± 1.98	2.28 ± 1.57		
			Winter period	1.87 ± 1.66	1.62 ± 1.33		
			*p* value	0.001	0.003		
			No statistical differences between summer and winter (*p* > 0.05) in suburban children.				
			Urban-traffic sites				
				MN (‰) (mean ± SD)	BEC with MN (‰) (mean ± SD)		
			Summer period	2.68 ± 1.99	2.68 ± 1.99		
			Winter period	1.64 ± 1.59	1.38 ± 1.15		
			*p* value	0.004	0.005		
			MN frequencies of urban-traffic children significantly higher in the summer than that of the winter (*p* < 0.05).				
Formaldehyde, nitrogen dioxide (NO2) in the air	Children 6–12 years old (living near chipboard-Viadana-Italy)/N = 413/oral mucosa cells	Comet assay MN assay	Children living near (<2 km) the chipboard industries—highest average exposure to formaldehyde.				[69]
			Comet assay		Mean		
			Tail intensity (%)		3.25		
			Tail lenght (µm)		11.69		
			Tail moment		0.20		
			Formaldehyde increase (0.20 µg/m^3^) associated with a 0.13% (95% CI: 0.03, 0.22%) higher comet tail intensity, 0.007 (95% CI: 0.001, 0.012) higher tail moment.				
			Micronuclei assay (%)				
			MN: 0.12				
			NBUDs: 0.23				
			NO_2_ increase (2.13 µg/m^3^) was associated with a 16% relative increase (RR = 1.16; 95% CI: 1.06, 1.26) in NBUDs.				
Heavy Metals: arsenic, chromium, lead, manganese, molybdenum, zinc	Adults (working in the Panasqueira mine or living in the same region)/N = 122/blood samples	Comet assay (% DNAT) MN assay		Controls	Environmentally exposed	*p*-value	[70]
				Mean	Mean		
			% DNAT	12.40	24.58	<0.001	
			MN (‰)	6.45	8.46	0.002	
Heavy metals	Adults (average age: 35.41) in 5 Bosnian regions with extensive mining, industrial activities/N = 104/blood samples	CBMN assay.	Frequencies—range and mean ± SD				[71]
			Total number of MN in BN cells: 1.00–27.00‰ and 8.35 ± 5.38.				
			MN: 0.10–2.50% and 0.83 ± 0.54.				
			NPB: 0.00–12.00‰ and 3.46 ± 2.89.				
			NBUD: 0.00–10.00‰ and 2.40 ± 2.22.				
			MN frequency (%) in BN cells no statistically significant differences between any of the studied group as compared to the control group (*p* > 0.05).				
			NPBs differences were found to be statistically significant between 3 regions as compared to the controls (*p* < 0.05), and NBUDs in the local population of 1 region as compared to the control group (*p* < 0.05).				
Herbicide (alachlor)	N = 1 male (age 43)/N = 1 female (age 30)/mononuclear isolated leukocytes	CBMN assay	The induction of MN-BN in isolated lymphocytes was not statistically significant (p = 0.18) although one of the replicates at the highest concentration (20 µg mL^−1^) was much higher than the other replicate, leading to a higher, but not statistically significant difference.				[72]
			Isolated blood lymphocytes				
			Alachlor [µg/mL]		MN (per 1000)		
			0.0		6.0 ± 0.0		
			2.5		6.0 ± 2.1		
			5.0		5.5 ± 0.7		
			10.0		6.8 ± 0.4		
			20.0		10.3 ± 4.6		
			Isolated human lymphocytes treated for last 51 h of a 72 h culture period.				
			Isolated human lymphocytes				
			Alachlor [µg/mL]		MN in BN cells (per 1000)		
			0.0		3.8 ± 0.4		
			2.5		4.8 ± 3.2		
			5.0		4.5 ± 0.7		
			10.0		4.8 ± 1.8		
			20.0		Too few dividing cells		
			40.0		Too few dividing cells		
			4 h treatment with alachlor				
			Alachlor [µg/mL]		MN in BN cells (per 1000)		
			0.0		6.5 ± 2.1		
			2.5		n.d.		
			5.0		n.d.		
			10.0		n.d.		
			20.0		4.5 ± 0.7		
			40.0		13.5 ± 3.5		
Mobile phone radiation	Male adults (age 20–30)/N = 300 (150 high mobile users and 150 low mobile users)/buccal epithelial cells	MN assay	Group I mean ± SD (0.77 ± 0.815).				[73]
			Group II mean ± SD (1.52 ± 1.176).				
			Significant increase in the mean MN count in group II in comparison to the group I (*p*-value < 0.0001).				
			In group II, the MN count in the side of mobile phone use was found to be statistically significantly elevated (1.52 ± 1.176) in comparison to the opposite side (0.90 ± 0.3992).				
			MN mean count was found to be significantly increased in non-head phone users (2.08 ± 1.291) in comparison to headphone users (0.96 ± 0.699).				
Pesticides (complex mixtures): carbamates, organophosphates, pyrethroids	N = 239 agricultural workers/N = 231 unexposed controls/lymphocytes of peripheral blood (PBL) and exfoliated cells of the oral mucosa	CBMN assay in PBL MN assay			Mean ± SE		[74]
			BNMN	Control	12.25 ± 0.60		
				Exposed	11.40 ± 0.49		
			MNL	Control	13.82 ± 0.69		
				Exposed	12.55 ± 0.55		
			BCMN	Control	1.06 ± 0.10		
				Exposed	1.03 ± 0.09		
			MNBC	Control	1.18 ± 0.12		
				Exposed	1.12 ± 0.10		
Pesticides environmental exposure (through inhalation): glyphosate, liquid formulations of cypermethrin, chlorpyrifos	Children (age 4–14)/N = 50 pesticide spraying areas (Córdoba)/N = 25 children from the city of Río Cuarto (Córdoba), not exposed to pesticides/buccal mucosa cells	MN assay	MN mean per 1000 cells Marcos Juárez: 5.20 ± 0.58 Río Cuarto: 3.36 ± 0.63 Genotoxicity is present in a group of children in Marcos Juárez was higher compared from to the Río Cuarto.				[75]
Pollution containing: cadmium, lead, p,p'-DDE, hexachlorobenzene, PCBs, dioxin-like t,t'-muconic acid, 1-hydroxypyrene	Adult residents (age 50–65) from 9 areas with different types of pollution/N = 1583/peripheral blood cells	MN assay Comet assay (% DNA)		MN mean	% DNA mean		[76]
			Antwerp	7.30	1.69		
			Antwerp port	6.65	1.23		
			Fruit area	6.00	1.35		
			Olen	7.00	1.60		
			Ghent	7.25	2.03		
			Waste incinerators	8.60	2.24		
			Rural area	7.00	1.97		
			Within an industrial area DNA strand break levels were almost three times higher close to industrial installations than 5 kilometres upwind of the main industrial installations (*p* < 0.0001).				
			Overall significant differences between areas were still observed for oxidative DNA damage (*p* = 0.040) and for DNA-strand breaks (*p* < 0.001) and for MN (*p* = 0.11).				
Polycyclic aromatic hydrocarbons (PAHs) in the air	Children (age: 6–15)/5 groups of Tabasco-Mexico 5 groups/peripheral blood lymphocytes	Comet assay		Exposed children	Control group		[77]
			Tail lenght	14.21–42.14	12.25		
			Tail/head	0.97–2.83	0.63		
PAHs and lead (Pb)	Children (age: 5–14), 2 most polluted cities-Katowice, Sosnowice/N = 74/peripheral blood lymphocytes	MN assay	MN mean: 4.44 Individual values reaching 17 MN cells per 1000 binucleated cells. Positive significant correlation was found between PbB and MN levels (r = 0.347, p < 0.05).				[78]
Pyrethroid insecticide	Males (age: 25–30)/N = 5/peripheral blood samples /human hepatoblastoma derived cell line HepG2	Alkaline comet assay with FPG	Dose dependent increase of DNA damage in both cell types, positive correlations between DNA damage in lymphocytes (tail DNA, *r* = 0.982, *p* > 0.001 and tail lenght, tail DNA, *r* = 0.957, *p* > 0.001. HepG2: tail DNA, *r* = 0.848, *p* < 0.05 and tail lenght, *r* = 0.848, *p* < 0.05.				[79]

## 6. Occupational Exposure

A wide range of chemicals that can act as environmental hazards, may also be exposure factors in specific occupational settings, and this is an extremely important consideration. For instance, besides the risks to the general public, atmospheric pollution can be considered an occupational health hazard to professional groups, such as traffic police or professional drivers working in urban areas [62], organic solvents [34], [80], [81], and others. Biomonitoring of exposure to toxic chemicals in the workplace is a fundamental tool to evaluate human health risks, supporting strategies to establish a safe work environment [82]–[85]. Table 2 summarizes some important occupational exposures, namely, antineoplastics [84], byproducts of petrol [85], formaldehyde [86], heavy metals [69],[87],[88], methyl bromide [89], organic solvents and smoke generated from biomass burning [34],[80],[81],[90]–[92].

Occupational risk assessment may be defined as the qualitative and quantitative characterization of an occupational risk, i.e., the probability that an adverse health effect may result from human exposure to a toxic agent which is present in the occupational setting. It has three fundamental tools: environmental monitoring, health surveillance and biological monitoring. Risk assessment is meant to quantify the likelihood that a quantitatively defined occupational exposure of an individual (or group of individuals) to a chemical might result in adverse health effects [14],[82].

National and international bodies set maximum allowable workplace concentrations for a wide range of substances. For instance, for airborne exposure to gases, vapors and particulates, recommended or mandatory occupational exposure limits (OELs) have been developed in many countries. The most widely used limits, called threshold limit values (TLVs), and are those issued in the United States of America by the American Conference of Governmental Industrial Hygienists (ACGIH). Specifically for airborne exposures, there are three other types of limit, namely the time-weighted average (TWA) exposure limit—the maximum average concentration of a chemical in air for a normal 8-hour working day and 40-hour week; the short-term exposure limit (STEL)—the maximum average concentration to which workers can be exposed for a short period (usually 15 minutes); and the ceiling value—the concentration that should not be exceeded at any time [83]. However, there is a need for revision of workplace limits to take also into account the levels of various agents that can cause allergies, for instance, in addition to occupational diseases. As new agents are identified they should be swiftly regulated.

**Table 2.** Studies of human populations related occupational exposures.

Risk factor/exposure	Studied population/number of samples/sample	Genotoxicity biomarkers	Results																						Refs.
Antineoplastics	Occupationally exposed nurses N= 27/N = 111 non-exposed subjects/peripheral blood cells	CBMN assay	MN lymphocytes mean ± SE (range)																						[84]
			Controls: 2.09 ± 0.312 (0–15)																						
			Exposed: 10.11 ± 2.053 (1–58)																						
			The occupationally exposed group showed significantly higher MN mean (*p* value < 0.001, Mann-Whitney test).																						
Benzene	Gasoline station attendants (GSA) N = 43/controls N = 28/whole blood, buccal exfoliated cells	Comet assay in whole blood MN assay in buccal exfoliated cells	DNA damage index, significant increase in the damage score in the GSA group compared to controls (Mann-Whitney test, *p* < 0.001). 3.8-fold higher in the GSA group compared to controls (Mann-Whitney test, *p* < 0.001).																						[81]
Benzene and atmospheric pollutants	Gas station attendants (GSA N = 43) taxi drivers (TD N = 34)/persons without known occupational exposures (NE N = 22)/buccal cells, blood	MN assay buccal cellsComet assay blood lymphocytes	Micronucleus assay																						[34]
			In the MN assay, no significant difference was observed among the groups (*p* > 0.05).																						
			Frequency of abnormal cells (MN/1000 cells):																						
			NE: 0.72																						
			GSA: 2.70																						
			TD: 1.30																						
			Comet assay																						
			Significant increase in DNA damage index (DI) in GSA and TD groups comparing to NE group (*p* < 0.001).																						
Byproducts of petrol and lead	Workers of car and battery repair garages N = 60/control group N = 80 workers who were not exposed to byproducts of petrol and lead/exfoliated cells of buccal mucosa	MN assay	MN mean (3000 cells per individual) Exposed: 8.22 Controls: 2.12 A significant difference (*p* < 0.001) was found between the exposed and the control.																						[85]
Formaldehyde	N = 46 workers occupationally exposed to formaldehyde (20–61 years old)/N = 85 unexposed individuals (20–53 years old)	CBMN assay in peripheral blood lymphocytes MN assay in buccal cells			MN in lymphocytes							NPB				NBUD					MN in buccal cells				[86]
					Mean							Mean				Mean					Mean				
			Controls		0.81							0.18				0.07					0.16				
			Exposed		3.96							3.04				0.98					0.96				
			All genotoxicity biomarkers showed significant increases in exposed workers in comparison with controls (Mann-Whitney test, *p* < 0.002).																						
Heavy metals: arsenic, lead, chromium, ma-nganese, moly-bdenum, zinc	Adults (workers in the Panasqueira/N = 122/blood samples	Comet assay (% DNA) MN assay		Controls									Occupationaly exposed							*p*-value					[69]
				Mean									Mean												
			% DNA	12.40									18.73							<0.001					
			MN (‰)	6.45									4.98							0.002					
			The occupationally exposed group showed significantly higher % DNA.																						
Heavy metals lead (Pb)	N = 90 male Pb recovery unit workers/N = 90 matched controls/peripheral blood lymphocytes, buccal exfoliated cells	Comet assay in PBL MN assay in buccal exfoliated cells and PBL	Comet assay																						[87]
													Comet tail lengh (µm)												
			Controls										8.15												
			Exposed										17.86												
			The results indicated that the exposed workers had a significantly higher mean comet tail length than that of controls (*p* < 0.05).																						
			Micronucleus assay																						
			MN frequency (‰)				Buccal cells										Lymphocytes								
			Controls				2.97										3.17								
			Exposed				4.66										6.46								
			Increased MN frequency in exposed subjects than in controls (*p* < 0.05).																						
Heavy metals: nickel chromium	N = 204 male subjects (age: 18–50) in India/N = 102 welders employed in welding plants, durations of exposure (1–24 years)/N = 102 subjects-control group/blood lymphocytes, buccal epithelial cells	Comet assay MN assay				Basal DNA damage (µm)								MN frequency (%)											[88]
						Mean				Range				Mean 0.32					Range						
			Control			8.94				4.14–17.10									0.00–0.80						
			Welders			23.05				17.24–35.62				1.30					0.12–2.89						
			The results indicated that the welders had a larger mean comet tail length than that of the controls (*p* < 0.001).																						
			Welders showed a significant increase in micronucleated cells compared with controls (*p* < 0.001).																						
Methyl bromide	N = 31 Methyl bromide-exposed fumigation workers/n = 27 referents/blood lymphocytes and oropharyngeal cells	Oropharyngeal MN assay (buccal cells) lymphocyte MN assay (blood lymphocytes)	MN assay (MN/1000 buccal cells) mean:																						[89]
			Workers: 2.00																						
			Referents: 1.31																						
			Two-sided *p*-value = 0.08.																						
			Kinetochore-negative micronucleated cells/1000 lymphocytes mean:																						
			Workers: 10.48																						
			Referents: 10.41																						
			Kinetochore-positive micronucleated cells/1000 lymphocytes mean:																						
			Workers: 10.81																						
			Referents: 10.44																						
			No statistically significant differences were observed between workers and referents for mean kinetochore-negative lymphocyte MN.																						
Organic solvent mixtures: acetone, 1-hexane, toluene, methylethylketone	N = 45 footwear industry workers: solvent based adhesive (SBA N = 29)/water solvent based adhesive (WSA N = 16)/N = 25 controls/blood, buccal cells	Comet assay CBMN assay									Control				WBA						SBA				[90]
			Comet assay (blood)																						
			Damage index								3.44 ± 3.24				2.13 ± 2.45						8.35 ± 7.85				
			Damage frequency (%)								1.52 ± 1.31				0.78 ± 0.91						2.76 ± 1.99				
			Micronucleus test																						
			MN (lymphocytes)								5.20 ± 2.33				3.88 ± 1.93						4.90 ± 2.34				
			NPB (lymphocytes)								3.00 ± 1.97				2.56 ± 2.53						3.69 ± 2.49				
			MN (exfoliated buccal cells)								0.62 ± 0.73				0.69 ± 0.87						1.15 ± 1.45				
			The Comet assay results showed that there was a significant increase in the mean damage index for the SBA (*p* < 0.001) group in comparison to the WBA group and control (*p* < 0.05).																						
			For the MN test in binucleated lymphocytes and exfoliated buccal cells, the 3 groups were not statistically different.																						
Smoke generated by biomass burning	N = 23 sugar cane workers/N = 30 control group/blood lymphocytes, buccal exfoliated cells	MN assay	Micronucleus assay (MN/1000 cells)																						[91]
									MN mean (lymphocytes)									MN mean (buccal cells)							
			Controls						1.27									9.70							
			Cutters						8.22									22.75							
			The MN frequencies in lymphocytes were higher (*p* < 0.001) in the sugar cane workers compared with the control group.																						
			A higher MN frequency in exfoliated cells was obtained in the group of sugar cane cutters compared with the controls (*p* < 0.001).																						
Toluene	N = 34 male industrial painters, occupationally exposed to toluene/N = 27 control group subjects with no history of occupational exposure/blood lymphocytes, buccal cells	Comet assay MN assay	Comet assay (DNA damage index):																						[80]
			Controls: 39.4																						
			Painters: 60.4																						
			Significant increase in DNA damage index between painters and controls (*p* < 0.001).																						
			Micronucleus assay (MN/1000 cells)																						
			Controls: 2.24																						
			Painters: 2.74																						
			No significant difference between painters and controls (*p* > 0.05).																						
	N = 34 women from shoemaking plants (n = 16 plant A + n = 18 plant B)/N = 19 controls/blood mononuclear lymphocytes	Comet assay						TM											% TDNA						[92]
			Controls					5.37 ± 2.48											18.18 ± 6.26						
			Workers plant A					5.85 ± 2.43											19.49 ± 5.80						
			Workers plant B					6.09 ± 1.91											20.26 ± 4.35						
Vehicle exhaust	N = 49 traffic police with outdoor activitiesN = 36 indoor workers from university/lymphocytes	CBMN assay						Mean ± S.D.											95% CI						[62]
			Controls					4.83 ± 1.84											4.20–5.46						
			Traffic police					7.06 ± 2.87											6.23–7.89						
			(*p* = 0.001, Wilcoxon test).																						

## 7. Diet

Dietary habits are recognized to be an important modifiable environmental factor influencing cancer risk and tumor development, and other diseases. Although some studies have estimated that about 30–40% of all cancers are related to dietary habits, the actual percentage is highly dependent on the foods consumed and the specific type of cancer [18],[93],[94]. Epidemiological studies on the role of environmental exposure to carcinogens in diet have identified specific cancers whose incidence is known to vary considerably among countries [89]; substantial increases in the risk of certain cancers are observed in populations migrating from low- to high-risk areas, and this suggests that international differences in cancer incidence can be attributed primarily to environmental or lifestyle rather than genetic factors [93],[95]. Diet can influence cancer development in several ways, namely by direct action of carcinogens in food that can damage DNA, by dietary components that can change enzyme activity, or by inadequate intake of molecules involved in antioxidant protection, DNA synthesis, repair or methylation that can influence mutation rate or changes in gene expression [96], and others. It is important to note, however, that the role of dietary components with potential cancer chemopreventive activity is not the subject of this review [3].

Another perspective of diet related to cancer risk is unintended contamination, which can result from compounds used in agriculture (e.g., pesticides and herbicides in plant-based foods, and growth hormones or antibiotics used in animal farming), or food processing (e.g., preservatives, smoking) and food packaging (e.g., bisphenol A or phthalates). The latter are not known to directly cause cancer, but they may influence cancer risk in other ways—for example, by acting as hormone-like substances in the body [97]. Is important to note that heavy metals, such as cadmium or mercury, may enter the food chain, such as in fish, or they may enter through contamination or their natural presence in soil or water.

Many substances are added to foods to prolong shelf and storage life and to enhance color, flavor, and texture. The possible role of food additives in cancer risk is an area of great public interest [97]. Briefly, food additive is a substance not normally consumed as food by itself and not normally used as a typical ingredient of the food, whether or not it has nutritive value [98].

The presence of such chemical contaminants or other unwanted substances in food and feed is often unavoidable as some of these substances are ubiquitous in the environment. However, the collection of dietary intake data along with chemical analysis of biological samples allows human biomonitoring programs to identify chemical exposures that might be associated with diet [60].

The European Food Safety Authority (EFSA)—commissioned project to review the state of the art of human biomonitoring for chemical substances and its application to human exposure assessment for food safety, facilitated the identification of vulnerable populations (e.g., by age, sex, socioeconomic status, etc.) as well as chemical exposure associated with food intake [60]. An important and specific context where the studies in diet have been raising more attention and concerns are maternal diet during pregnancy, this being the main source of essential nutrients that are needed for optimal fetal and child development. This applies no just to diet itself but also to prenatal exposure to several environmental pollutants which enter the mother's body as food contaminants, such as dioxins, PAHs and polychlorinated biphenyls [99],[100].

**Table 3.** Studies of human populations related dietary exposures.

Risk factor/exposure	Studied population/number of samples/sample	Genotoxicity biomarkers	Results			Refs.
Arsenic Cooked rice with > 200 µg/kg	Adults not significantly exposed to arsenic through drinking water (west Bengal-India)/N = 400/urothelial cells	MN assay		MN range	MN mean	[101]
			Whole cohort cooked rice arsenic (µg/kg)	0.50–4.98	2.12	
			Lowest cooked rise arsenic group ≤ 100		1.85	
			Highest cooked rice arsenic group > 300		3.23	
			Groups with mean cooked rice arsenic > 200 µg have significantly higher (*p* < 0.05) induction of genetic damage compared to each of the groups with mean cooked rice arsenic ≤ 200 µg/kg.			
Beauvericin and ochratoxin A	N = 1 female (age: 50)/human leukocytes PK15 cells	Comet assay	BEA (0.5 µM) and OTA (1 and 5 µM) as well as all toxin combinations produced a significant increase in tail moment compared to control cells (*p* < 0.05). BEA alone at either concentration had a significantly lower DNA damage than BEA and OTA combinations (*p* < 0.05).			[102]
Food additive benzoic acid	N = 2 adults (age: 24–25)/human peripheral blood lymphocytes	MN assay	Benzoic acid significantly increased micronucleus frequency (200 and 500 µg/mL). This increase was dose-dependent (*r* = 0.79).			[103]
Monosodium glutamate (MSG)	N = 3 adults (age: 23–26)/peripheral blood samples	CBMN assay Comet assay	MN assay:			[58]
			Increase dose dependent (*r* = 0.96).			
			Comet assay:			
			% Tail intensity: *r* = 0.60.			
			Mean tail lenght (mm): *r* = 0.59.			
			Tail moment: *r* = 0.71.			
			Increase dose dependent.			
Sodium sorbate (SS)	N = 2 adults (age: 24–25)/peripheral blood	MN assay Comet assay	SS increased SCEs/cell and MN frequency at 400 µg/mL and 800 µg/mL concentrations at both 24 h and 48 h compared to negative control.			[104]
			Comet assay	Average tail intensity (%)		
			Negative control (c = 0 µg/mL)	2.73		
			SS (c = 400 µg/mL)	10.91		
			SS (c = 8000 µg/mL)	5.97		
			SS is genotoxic to the human peripheral blood lymphocytes in vitro at the highest concentrations.			
Synthetic food colorants Sunset yellow FCF and brilliant blue FCF	N = 10 adults/blood samples.	MN assay	MN frequency was increased with increasing concentrations of sunset yellow and brilliant blue.			[105]
			Sunset yellow, significant increases in the MN rates were detected 30 mg/mL and 40 mg/mL of the concentrations (*p* < 0.05).			
			Brilliant blue, significant increases in the MN rates were detected 30 mg/mL and 40 mg/mL of the concentrations (*p* < 0.05).			
Erythrosine (E127), tartrazine (E102), ponceau 4R (E124), sunset yellow (E110), brilliant blue FCF (E133), fast green (E143), carmoisine (E122), and indigo carmine (E132)	N = 1 adult/blood samples.	CBMN assay	Statistically significant increase in MN means induced by various food colors (multivariate analysis, *p* = 0.001 and pairwise comparisons, *p* < 0.05). Control = 10 100µg/mL = 12 ± 0.7 200µg/mL = 12.8 ± 0.8 300µg/mL = 13.7 ± 0.7			[98]

Table 3 summarizes some important studies in diet field, namely the exposure to arsenic [101], mycotoxins as contaminants in food items [102], food additives [103],[104], flavor enhancers [58], and synthetic food colorants [98],[105].

For many other compounds for which the effects on cancer risk are not clear, there may be other good reasons to limit exposure. But at the levels that these are found in the food supply, lowering cancer risk is unlikely to be a major reason to justify this. There are moves to redefine maximum permissible limits for food colorants, instead of setting arbitrary limits for food additives in general; for instance in the case of colorants, each dye should have an individual limit based on well controlled genetic studies [98].

## 8. Conclusions

Human biomonitoring is a scientifically-developed approach for assessing human exposures to natural and synthetic compounds from the environment, occupation, and lifestyle, including diet [56]. It is the only available tool to integrate exposures from all sources and provide data for epidemiological studies of strengths of associations, dose response relations, etc.; however, it does not differentiate the exposure by source. Furthermore, human biomonitoring alone cannot provide information on how long a chemical has been in the body. Additional data collected from questionnaires, interviews and exposure assessment, combined with background knowledge, may provide valuable information regarding sources [21],[60].

Although there has been growing recognition for the need to incorporate complex interactions between environmental exposures together with genetic factors, in order to fully understand cancer and diseases causation, since genetic instability is the startup point of carcinogenesis, there is growing recognition that environmental challenges not only interact with genes but may also modulate genetic effects and influence phenotypes [106]. An optimistic message is the fact that cancer development is not an inevitable consequence of the aging process *per se*, although there is a partly avoidable increased likelihood of the requisite number of mutations occurred, and the human species is not inevitably destined to suffer a high incidence of cancer. This awareness has lent greater urgency to the search for more powerful tools for primary prevention, for early warning systems to identify causal environmental agents and flag risks well before a disease condition develops [5].

In conclusion, the potential benefits of biomarkers and molecular epidemiology in illness prevention justify a major commitment to the further development of human biomonitoring programs, the only available tool that combines exposure assessment from different sources and relates their effects, together with individual susceptibility, to the risk of disease.
